# The Economic Burden of Stroke Based on South Korea’s National Health Insurance Claims Database

**DOI:** 10.15171/ijhpm.2018.42

**Published:** 2018-05-07

**Authors:** Yu-Jin Cha

**Affiliations:** Department of Occupational Therapy, Semyung University, Jecheon, Republic of Korea.

**Keywords:** Cost of Illness, National Health Insurance, Economic Burden, South Korea, Stroke

## Abstract

**Background:** This study was conducted to determine the scale and the nature of the economic burden caused by strokes and to use the results as an evidential source for determining the allocation of South Korea stroke cases in 2015.

**Methods:** For research subjects, the study analyzed demographic characteristics and economic burden based on data from national health insurance (NHI) claims for inpatient and outpatient cases of ischemic stroke and hemorrhagic stroke in 2015 through the Health Insurance Review and Assessment Service (HIRA) and statistical data regarding cause of death from the Korea National Statistical Office (KNSO). This study, in order to estimate economic burden due to stroke, deduced the direct and indirect costs of illness caused by stroke, using cost-of-illness (COI) methods. The economic burden is divided into direct and indirect costs, and indirect cost is estimated by summing lost productivity measured in opportunity cost lost by medical disposition due to a specific disease and lost productivity due to premature death.

**Results:** The total economic burden in Korea due to stroke was US$6.855 billion, that due to ischemic stroke was US$3.658 billion, and that due to hemorrhagic stroke was US$3.197 billion. The average economic burden per stroke case was about US$7247.

**Conclusion:** The results of estimating the annual economic burden in all of Korea due to stroke will be used as an evidential source for preparing medical insurance policies, priorities, and plans for arranging medical resources for stroke as well as for determining effective prevention of the disease and related priorities in national health care policies.

## Background

Stroke is a major disease as it is the third cause of death following cancer and heart diseases in Korea, and has been a leading cause of death in developed countries such as the United States and European countries.^1^ Two-thirds of patients survive but most of them have serious disabilities exceeding middle levels, requiring comprehensive rehabilitation therapies for recovery from neurological damage and loss of function. Stroke is a leading cause of long-term disabilities, and only one quarter of patients recover sufficiently to lead independent lives.^2^ As a result, about a half of patients become a burden to their families or society within one year.^3^

In order to manage higher death rates and economic costs related to stroke, studies on cost-of-illness (COI) are ongoing in European countries and the United States. Economic COI studies are aimed at making policies for the distribution of resources used for preventing and treating diseases by suggesting medical costs used for treating diseases, transportation costs, care costs, disease morbidity costs, and productivity damage costs due to premature death with monetary units and representing effects of diseases on the national economy including individuals objectively and empirically.^4^

In 2013 the National Health Insurance Service (NHIS) of Korea established a standard big data database for researchers utilizing information regarding the history of treatments, health examinations, residence and premiums, and healthcare institutions. This consists of a standard cohort database for the population of a million, three databases related to rare diseases, and a database about health examinations. This data is offered for studies with public benefit. In Korea, the entire citizenry is insured under this single service, and a single insurance claim database was established, providing very good conditions for conducting COI of various diseases including stroke.^1^

In the case of the United States, more than US$22.8 billion was estimated as total annual direct costs due to stroke in the single year of 2009 when the rate of medical disposition related to the diseases was compared and measured.^5^ Studies from Sweden, Canada, the Netherlands, the United Kingdom, and New Zealand using data regarding the registration of patients into government systems, their management, and claims to insurance, along with recent studies from Germany and Japan estimating the burden of disease for stroke have been published.^1^

Korean studies of COI, were made by Lim et al, regarding the estimation of the economic cost of stroke cases using data from claims to the Health Insurance Review and Assessment Service (HIRA), the single point in 2005, a decade ago.^1^ In Korea after 10 years, now, with dramatic aging and increasing occurrence of stroke, many patients have participated in rehabilitation therapies. However, compared to other fields, there have been few studies regarding the estimation of the economic cost of the medical disposition of stroke cases. Accordingly, it is necessary to conduct holistic studies regarding social costs and the scale used for the costs of medication, inpatient stays, outpatient visits, rehabilitation, nursing home and so forth with regards to stroke.

## Methods

### Selection of the Research Subjects and Definition of the Variables

As research data to estimate the economic cost of stroke, insurance claim data generated during 2015 in Korea showing for the number of inpatient or outpatient visits to medical institutions were used. The study analyzed demographic characteristics and economic cost based on the data from National Health Insurance (NHI) claims regarding inpatient or outpatient treatment where, among ICD-Codes, ischemic stroke (International Classification of Disease-10-Clinical Modification I63) (ICD-10-CM I63) and hemorrhagic stroke (ICD-10-CM I61) were indicated as the main diagnoses in 2015 cases by HIRA, and the statistical data regarding stroke as cause of death (Korean Standard Classification of Diseases, KCD I60-I69) by the Korea National Statistical Office (KNSO).

In this study, in keeping with previous studies, the economic cost of stroke was divided into direct and indirect costs. The estimated direct medical cost consisted of outpatient visit cost, inpatient cost, medication cost, the cost for using the pharmacy, and assistive device cost while direct non-medical cost consisted of transportation cost and caregiver cost. Indirect cost included costs due to work loss and premature death.^4,6-9^

### Selection of Research Data

This study, estimated economic cost due to stroke using a COI method. Economic cost is divided into direct and indirect costs,^4^ and indirect cost is estimated by summing lost productivity measured in opportunity cost lost by medical disposition due to a specific disease and lost productivity due to premature death.^10,11^ All the analysis regarding inpatient and outpatient stroke-related treatment is based on data from 2015 (Supplementary file 1).

### Statistical Analysis

The data analysis in the study was done by using SPSS 12.0, and frequency analysis was conducted. The analysis results were calculated using Excel.

### Estimation of Direct Costs

In this study, treatment cost, one of the direct costs includes inpatient and outpatient costs analyzed according to age and gender for the appropriate disease codes (ICD-10-CM I61 and I63) in the main statistical data for 2015 at the NHI Corporation, with total treatment costs estimated by adding covered inpatient (outpatient) treatment costs and non-covered inpatient (outpatient) treatment costs. Treatment costs recorded by NHI do not include the non-covered allotment paid by the individual but include the portion covered by NHI after the legally allotted portion is paid by the individual. Therefore, the non-covered allotment paid by the individual was estimated separately by applying the non-covered allotment paid by the individual for inpatient and outpatient treatment during the year using research on current treatment costs for insured patients as seen below.

To estimate the cost of assistive devices for stroke cases, the annual cost for a patient to keep an assistive device was multiplied by the number of stroke patients. The annual cost for a patient to keep an assistive device was determined by adopting the cost for 2010 indicated in Kim’s study, 241 512 KRW (US$206) which was adjusted using the consumer price inflation rate,^12^ to determine a 2015 value, and that amount was multiplied by the number of stroke patients to estimate the cost. The total direct costs can be estimated by summing inpatient and outpatient treatment costs, medication costs and costs for using the pharmacy, costs for buying and keeping assistive devices, transportation costs, and caregiver costs.

Direct non-medical cost is divided into transportation cost used for outpatient visits and caregiver cost. Transportation costs were calculated for outpatient treatments by multiplying the number of claims for outpatient visits by an average round-trip rate of 21 000 KRW per visit to determine the total estimated transportation cost.^7,13^

For caregiver cost for inpatients, the average daily caregiver cost cited in the Intermediate Status of Model Project for Institutionalization of Hospital Caregiver Service by the Ministry of Health and Welfare (2010), the most recent data available was adjusted using the consumer price inflation rate to convert it to a 2015 value, and then it was multiplied by the number of inpatient days to estimate the total amount.^7^

### Estimation of Indirect Cost

Indirect cost measures lost opportunity cost due to medical disposition or premature death and consists of the indirect COI due to loss of productivity and the indirect COI due to premature death.^3^ The former includes reductions in work income for lost hours or days due to outpatient visits or hospitalization related to diagnosis and treatment of a disease. Outpatient visits result in a different amount of lost work time from hospitalization, so the total cost was arrived at by calculating the two separately and adding them together. If a person has two outpatient visits in one day, they were counted as two separate visits. One outpatient visit was assumed to require one-third of a work day. Considering one outpatient visit as equivalent to 1/3 of a day with regard to lost productivity, three outpatient visits were same as one day’s hospitalization.^13^

### Indirect Cost of Lost Productivity

For the calculation of cost due to premature death, after calculating the number of deaths due to stroke by age, life expectancy of stroke cases (lost years),^14^ average annual expected income, and employment rate were multiplied to estimate the total cost. In these calculations, the ages between 1 and 19 and after 70 were considered to have no income (Supplementary file 2).^13^

### Costs Per Stroke Event

The average economic cost per stroke case was calculated by dividing the total cost excluding the cost of premature death by the number of patients, with the following estimation formula is as follows^1^ :

## Results

### The Characteristics and Current Status of the Research Subjects

For the period of the analysis, from January 1, 2015 to December 31, 2015, the numbers of stroke events resulting in treatment who were treated in medical facilities in Korea covered by NHI was 515 848, 1.05% of the 49 088 000 total treated patients claimed for review and assessment in 2015. Their demographic features by gender, age and medical facility are as follows (Table 1). Gender, age, and ICD-10 show the number of stroke events, and disposition and medical facility show the number of visits. The gender distribution of those diagnosed as having suffered a stroke, included 277 641 male patients (53.82%) and 238 207 female patients (46.18%). With regard to the distribution of occurrence of stroke by age group, the highest share was for the ages from 70 to 79, with 178 504 patients (34.60%). Regarding disposition, outpatient visits cases were 472 371 (78.48%), and hospitalization cases were 129 513 (21.52%), the former being about 3.6 times as many as the latter. With regards to primary medical facilities used in the treatment of stroke cases, general hospitals were used by a total of 232 450 patients (39.54%), and tertiary hospitals by 187 263 patients (31.85%). In comparing the frequency of occurrence, ischemic stroke occurred in 463 147 patients (89.78%), about 8.8 times as many as the 52 701 patients (10.22%) with haemorrhage stroke. ‘Hospitalization’ is the number of inpatient visits. If a patient made two inpatient visits in 2015, does ‘hospitalization’ count them as two. The medical facility is the same. One patient may visit two or more different types of medical facility. Some patients may be duplicated in the disposition and medical facility variables.

**Table 1:** General Characteristics of Stroke Case in 2015 (N = 515 848)

**Variables**	**Category**	**Stroke cases**	
		No.	%
Gender	Male	277 641	46.18
	Female	238 207	53.82
Age	0-9	657	0.13
	10-19	575	0.11
	20-29	1465	0.28
	30-39	5558	1.08
	40-49	24 243	4.70
	50-59	78 878	15.29
	60-69	128 542	24.92
	70-79	178 504	34.60
	80+	97 426	18.89
Disposition^a^	Hospitalization	129 513	21.52
	Outpatient visits	472 371	78.48
Medical facility^a^	Tertiary hospital	187 263	31.85
	General hospital	232 450	39.54
	Hospital	102 535	17.44
	Clinic	63 863	10.86
	Public health center, etc	1833	0.31
ICD-10	Ischemic stroke (163)	463 147	89.78
	Hemorrhagic stroke (161)	52 701	10.22

Abbreviation: ICD-10, International Classification of Disease-10 version. ^a^ Some patients may be duplicated.

### Estimation of South Korea Economic Cost Due to Stroke

In 2015, Korea’s economic cost due to stroke was 7.4728 trillion KRW (US$6.855 billion): That for ischemic stroke was 3.9874 trillion KRW (US$3.658 billion) while that for hemorrhage stroke was 3.4853 trillion KRW (US$3.197 billion). The economic cost for ischemic stroke was 53.36% of the total stroke cost, and 502.0 billion KRW (US$460 million) more than that for hemorrhagic stroke. Among the total economic costs due to stroke, the share of lost productivity due to premature death was 45.53% with the highest portion, followed by inpatient/outpatient treatment cost (26.93%), loss of work (10.73%), and medication cost (5.09%) (Table 2).

**Table 2:** Economic Costs of Stroke in South Korea, 2015 – Unit*:* 1 million won (%)

**Cost items**	**Subcategory**	**Ischemic Stroke**	**%**	**Hemorrhagic Stroke**	**%**	**Stroke**	**%**
Direct costs	Direct medical costs (1)	Inpatient costs	1 378 782	34.58	519 458	14.90	1 898 241	25.40
		Outpatient costs	99 382	2.49	14 903	0.43	114 285	1.53
		Non-covered care costs	263 113	6.60	91 375	2.62	354 489	4.74
		Medication costs	335 280	8.41	45 409	1.30	380 689	5.09
		Assistive devices costs	122 270	3.07	13 913	0.40	136 183	1.82
		Total medical costs (1)	2 198 827	55.15	685 058	19.65	2 883 887	38.58
	Direct non-medical costs (2)	Transportation costs	57 387	1.44	8924	0.26	66 312	0.89
		Caregiver’s costs	242 573	6.08	75 720	2.17	318 293	4.26
		Total direct non-medical costs (2)	299 961	7.52	84 644	2.43	384 606	5.15
	Total direct costs (1) + (2)		2 498 788	62.67	769 702	22.08	3 268 493	43.73
Indirect costs	Lost productivity due to premature death (3)		881 203	22.10	2 521 126	72.33	3 402 329	45.53
	ost productivity due to hospitalization and outpatient visits (4)		607 442	15.23	194 562	5.58	802 005	10.73
	Total indirect costs (3) + (4)		1 488 646	37.33	2 715 689	77.91	4 204 334	56.26
**Total costs (1) + (2) + (3) + (4)**			**3 987 434**	**100**	**3 485 391**	**100**	**7 472 828**	**100**

### Direct Cost

Direct costs include inpatient cost, outpatient cost, non-covered care cost, medication cost and cost for using the pharmacy, and the cost of assistive devices cost and the total direct cost for stroke was 32 684 trillion KRW (US$2.998 billion): For ischemic stroke, it was 2.4987 trillion KRW (US$2.292 billion) while it was 769.7 billion KRW (US$706 million) for hemorrhagic stroke. The direct medical cost for stroke was 2.8838 trillion KRW (US$2.645 billion): For ischemic stroke, it was 2.1988 trillion KRW (US$2.017 million) while it was 685.0 billion KRW (US$628 million) for hemorrhage stroke; the former was about 3.2 times higher. The direct non-medical cost for stroke was 384.6 billion KRW (US$320 million): For ischemic stroke, it was 299.9 billion KRW (US$257 million) while it was 84.6 billion KRW (US$72 million) for hemorrhage stroke, the former being around 3.6 times higher. Direct cost represented 43.73% of the total economic cost for stroke. Inpatient and outpatient cost was 26.93% with the highest share (Table 2).

### Indirect Cost

Indirect cost is the sum of the losses due to lost work and premature death, and the indirect cost for stroke was 4.2043 trillion KRW (US$3.857 billion): For ischemic stroke, it was 1.4886 trillion KRW (US$1.365 billion) while for hemorrhagic stroke it was 2.7156 trillion KRW (US$2.491 billion), 1.8 higher than that for ischemic stroke. For indirect cost due to stroke, loss of income was 3.4023 trillion KRW (US$3121 million), loss of work was 802 billion KRW (US$687 million), respectively, with income loss due to premature death was 4.5 times higher (Table 2).

### Estimation of Costs Per Stroke Event

The average economic cost per stroke case was about 7.9 million KRW (US$7247): While the cost for ischemic stroke cases was 6.7 million KRW (US$6146), the cost for hemorrhage stroke cases was 18.5 million KRW (US$16 972), 2.8 times as much as that in ischemic stroke cases (Table 3).

**Table 3:** Costs Per Stroke Event, 2015

**Detailed Items**	**Ischemic Stroke**	**Hemorrhage Stroke**	**Stroke**
Costs per stroke case (1 million kwn)	6.7	18.5	7.9

Abbreviation: kwn, Korean won.

## Discussion

This study was conducted to estimate the scale and the nature of economic damage due to stroke with the intention that it be used as an evidential source for determining the efficient allocation of limited medical resources, and to support the development of policies concerning the prevention and management of the disease by estimating the scope of the economic cost of South Korea stroke cases in 2015, and an average annual economic cost per stroke case.

For patients with stroke treated in South Korea medical facilities using NHI in 2015, male patients outnumbered female patients, and the 70 to 79 age group ranked highest. Regarding disposition, the outpatient cases were about 3.6 times more frequent than those requiring hospitalization. For primary medical facilities used by the patients, general hospitals showed the highest share, followed by advanced general hospitals and then standard hospitals followed in order. The type of stroke that occurred most frequently was ischemic stroke, about 8.8 times more often than hemorrhagic stroke. About 71% of all stroke cases use tertiary and general hospitals in Korea, compared to the case of Australia where hospitals and nursing homes are primarily used.^15^ In the majority of the developed countries, the demand for acute care beds is decreasing while demands for long-term care are greatly increasing, while the situation in Korea is different from other countries.^16^

In Korea, demands for long-term care is also dramatically increasing without requiring active treatment, and despite the preference in South Korea for hospitalized care even in cases where outpatient disposition or nursing home care would be sufficient, and the lack of long-term care facilities, the costs of using the facilities appear low. Therefore, in order to establish policies related to stroke in Korea, it is necessary to take measures to reduce the burden of cost due to unnecessary hospitalization through the rearrangement of medical personnel and improvement of facilities.^17^

Among the entire economic burden due to stroke, income loss due to premature death was highest, followed, in order, by the cost for inpatient and outpatient care, the cost for work loss, cost for medication, and the cost for using pharmacies. By examining the differences in allocation of cost for each type of stroke as identified by the study, recommendations for the allocation of medical resources to address stroke can be made. In the case of hemorrhagic stroke with a large share of the premature death cost, the lethality rate was higher while the direct medical costs represented a high portion of the cost. For ischemic stroke, the lethality rate was lower, but the treatment required high-priced medical devices and procedures. This implies that a different strategy is required to reduce economic burden depending on whether it is hemorrhagic or ischemic stroke that is being addressed.

In Kang et al,^18^ which examined the calculation of the medical cost of stroke in Korea in 2004, for brain hemorrhage inpatient cost was 48.06 billion KRW (US$42 million) and outpatient cost was 6.16 billion KRW (US$5 million), a total sum of 54.24 billion KRW (US$47 million). For ischemic stroke, inpatient cost was 135.9 billion KRW (US$118 million) and outpatient cost was 94.68 billion KRW (US$82 million), a total sum of 230.62 billion KRW (US$201 million). This figure represented 0.04% of the Korean gross domestic product (GDP) of US$680 billion in 2004. In Lim et al,^1^ about the estimation of COI for strokes occurring in 2005, the economic burden was about 3.7370 trillion KRW (US$3.267 billion) and the medical cost was about 1.1218 trillion KRW (US$980 million), 2.2% of the total healthcare cost in Korea in 2005. Ten years later, in 2015, Korean GDP was 1.5586 quadrillion KRW (US$1.336 trillion), and the 7.4728 trillion KRW (US$6.855 million), economic burden for stroke was 0.48% of the Korean GDP, and stroke treatment costs represented 13.77% of total medical costs covered by healthcare, 58.170 trillion KRW (US$49.787 billion). As a result, despite the differences in the monetary value and healthcare situations in 2015 from those about 10 years earlier, stroke has become a huge economic burden.

The reason was ischemic stroke cases, which numbered 8.8 times more than hemorrhagic stroke cases. Direct costs were 43.73% of the total economic burden and indirect costs were 56.26%. The reason that Korea has lower direct costs was that the costs of medical insurance were lower, causing a relatively lower share of direct costs.^19^ This implies that hemorrhagic stroke, in spite of lower morbidity, is a disease with the greater economic burden for the individual, and the priority in the prevention of stroke can be different depending whether the subjects are the entire nation or individuals.

In the study about cost according to use of resources by patients registered in the department of neurology in Philipps-Universität Marburg, Germany, the cost per patient was 3020 euros (US$3229) for ischemic stroke and 5080 euros (US$5432) for hemorrhage stroke. When including the cost of inpatient and rehabilitation after acute period, the cost was 9880 euros (US$10 565) per patient. In a Taiwanese study, using the results of research on direct costs for 360 patients for treatment of their first ischemic stroke with acute origin, the length of stay (LOS) was 7 days (range: 1-121 days), and the cost per patient was 26 326 NTD (New Taiwan Dollars, US$841). The amount spent per day for one patient was 3777 NTD (US$121).^20^

In this study, comparison with other countries in the medical disposition of stroke case is difficult, as a matter of fact. This is because of differences in demographic structure, the occurrence of stroke, and the compensation system are different, and these factors affect patients’ medical disposition and the status of medical suppliers’ use of medical and non-medical resources. For comparison, it is necessary to have standardized results about the status of patients, the current status of use of medical resources by type, and their effects.^17^

In Korea, the systematic medical rehabilitation system for stroke patients is still insufficient.^21^ The introduction and application of systems such as stroke units (SU) and early supported discharge (ESD) are necessary to reduce costs and improve resource utilization. In SU, an organized hospitalized service, teams from diverse disciplines are provided. There has been an increasing body of evidence suggesting the efficacy of SU in reducing mortality and morbidity.^22^ ESD is a system of the early discharge of mild and moderate stroke patients to a rehabilitation hospital in the community as soon as possible under government supervision.^23^ ESD has the benefit of minimizing the financial burdens on of patients and society while also bringing medical benefits.^24^

Regarding the limitations of the study; first, costs related to recreation and psychological pains were not included. Recently, in addition to direct costs, various indirect costs such as the cost of purchasing assistive devices, modifying housing facilities, and lost productivity of a patient, or patient’s care-giving family member, and other caregiver costs are being taken into consideration.^25^ Second, the cost of using long-term care insurance for the stroke patients was not included, and then the cost may be underestimated. In future studies, by including long-term care insurance service for the aged used by patients who have suffered strokes, it is expected that more accurate estimation can be made. Third, this study used the employment rate statistics of the whole population, not those of stroke cases. Fourth, it is thought that the indirect cost is less than the actual cost. This is because the life expectancy of the stroke patients decreases according to the disability level, which was not reflected in the productivity loss. Fifth, the data used in this study are limited in that secondary or tertiary diagnostics cannot be confirmed. Finally, there is a limitation to this study as it addresses not lifetime cost but annual costs. Also, it does not include all stroke survivors but those who utilize the health service, which can be listed in the insurance claim data.^26^ Thus, it is not a prevalence, nor incidence-based data.

## Conclusion

In Korea, the entire citizenry is insured by national health service, and a single base of insurance claim data has been established, which provides very good conditions for conducting studies to estimate COI. This study estimated the scale of the economic burdens for stroke cases in Korea, and the average annual economic burden per stroke case, and is expected to provide an evidential source for use in determining policies on insurance for the disease and for setting priorities and making plans for arranging medical sources as well as to determine priorities for the effective prevention of stroke and national healthcare policies.

## Acknowledgements

This paper was supported by the Semyung University, Jecheon, Republic of Korea Research Grant of 2016.

## Ethical issues

This study was exempted from the requirement for approval by the responsible ethics committee because it was a retrospective study of anonymized medical records.

## Competing interests

Author declares that he has no competing interests.

## Author’s contribution

YJC is the single author of the paper.

## Supplementary files

Supplementary file 1. Cost Classification and Data Sources of Economic Burden.

Supplementary file 2. Estimation of Indirect Cost of Lost Productivity Due to Premature Death by Age.

## Key messages

**Implications for policy makers**

- Determination of the scale and content of economic damage caused by stroke.
- The type of stroke occurring most frequently was ischemic stroke, at a rate about 8.8 times higher than that of hemorrhagic stroke.
- The economic burden in Korea due to stroke was US$6.855 billion.
- The average economic burden per stroke was about US$7247.
- It is expected that this data will be highly useful as an evidential source for preparing medical insurance policies, priorities, and plans.

**Implications for the public**

This study was conducted to determine the scale and the nature of the economic cost of stroke cases in South Korea in 2015. The aim of this study was to determine the economic costs associated with strokes suffered by South Korean patients from January 1, 2015 until December 31, 2015. The economic cost in Korea due to stroke was USD$6.855 billion, of which the cost of ischemic strokes was US$3.658 billion, and that of hemorrhagic strokes was US$3.197 billion. As a result of producing an estimate of the annual economic cost in the whole nation of Korea due to stroke, it is expected that this data will be highly useful as an evidential source for preparing medical insurance policies, priorities, and plans for arranging medical resources related to stroke.
