# Supplementary material for: The Economic Burden of Stroke Based on South Korea’s National Health Insurance Claims Database
Source: Int J Health Policy Manag. 2018 May 7;7(10):904–9. doi: 10.15171/ijhpm.2018.42 (PMC6186466; doi:10.15171/ijhpm.2018.42)
Supplement: Supplementary file 1 — Cost Classification and Data Sources of Economic Burden. [file ijhpm-7-904-s001.pdf]

Supplementary file 1. Cost Classification and Data Sources of Economic Burden [*Unit:* number, years, %, 1,000 kwn]

**Cost items**			**Data Sources**	**Variables**	
Direct costs	Direct medical costs	Inpatient costs	Main Statistical Data by NHI (2015)	Total Inpatient Treatment Cost for Ischemic Stroke	1,378,782,325 kwn
				Total Inpatient Treatment Cost for Hemorrhage Stroke	519,458,841 kwn
		Outpatient visit costs	Main Statistical Data by NHI (2015)	Total Outpatient Visit Treatment Cost for Ischemic Stroke	99,382,300 kwn
				Total Outpatient Visit Treatment Cost for Hemorrhage Stroke	14,903,319 kwn
		Non-cover care costs	Investigation on the Current Status of Treatment Cost for Health Insured Patients by NHI (2014)	Non-covered Patient's Burden Rate	17.1 %
			Consumer Price Research by KNSO (2014-2015)	Inflation Rate	0.7 %
		Medication costs	Main Statistical Data by NHI (2015)	Total Medication Cost and Cost for using Pharmacy for Ischemic Stroke	335,280,067 kwn
				Total Medication Cost and Cost for using Pharmacy for Hemorrhage Stroke	45,409,058 kwn
		Assistive devices costs	Assistive Devices Cost in Kim's study (2010)	Assistive Device Costs	241 kwn
			Consumer Price Research by KNSO (2010-2015)	Inflation Rate	9.5 %
			Main Statistical Data by NHI (2015)	Number of Patients of Ischemic Stroke	463,147 No.
				Number of Patients of Hemorrhage Stroke	52,701 No.
	Direct non-medical costs	Transportation costs	Family Economy Trend Research by KNSO (2015)	Average Transportation Cost for Round-Trip	21 kwn
			Main Statistical Data by NHI (2015)	Total Number of Claims for Ischemic Stroke Impatient	308,500 cases
				Total Number of Claims for Hemorrhage Stroke Impatient	88,920 cases
				Total Number of Claims for Ischemic Stroke Outpatient Visit	2,056,429 cases
				Total Number of Claims for Hemorrhage Stroke Outpatient Visit	237,909 cases

	Caregiver's costs	Intermediate Status of Model Project for Institutionalization of Hospital Caregiver Service by the Ministry of Health and Welfare (2010)	Average Daily Caregiver's Cost	33 kwn
		Main Statistical Data by NHI (2015)	Inpatient Days for Ischemic Stroke	6,612,878 days
			Inpatient Days for Hemorrhage Stroke	2,064,235 days
Indirect costs	Lost productivity due to premature death	The Statistical Data of Cause of Death by KNSO (2015)	Number of Patients who Died of Stroke	275,895 No.
		Kim's study about Cohort of Death of Patients of Stroke (2015)	Rate of Death by Ischemic Stroke	25.9 %
			Rate of Death by Hemorrhage Stroke	74.1 %
		Life Table by KNSO (2015)	Life Expectancy	82.06 years
		Employment, Work, and Income by KNSO (2015)	Average Annual Expected Income	39,600 kwn
			Employment Rate	62.6 %
	Lost productivity due to hospitalization and outpatient visits	Main Statistical Data by NHI (2015)	Outpatient visits of Patient of Ischemic Stroke	2,054,188 days
			Outpatient visits of Patient of Hemorrhage Stroke	237,695 days
		Employment, Work, and Income by KNSO (2015)	Average Daily Income	145 kwn

Abbreviation: KNSO, Korea National Statistical Office.
