# Supplementary material for: The Economic Burden of Stroke Based on South Korea’s National Health Insurance Claims Database
Source: Int J Health Policy Manag. 2018 May 7;7(10):904–9. doi: 10.15171/ijhpm.2018.42 (PMC6186466; doi:10.15171/ijhpm.2018.42)
Supplement: Supplementary file 2 — Estimation of Indirect Cost of Lost Productivity Due to Premature Death by Age. [file ijhpm-7-904-s002.pdf]

Supplementary file 2. Estimation of Indirect Cost of Lost Productivity due to Premature Death by Age

Age (y)	Year of Loss (Life Expectancy 82.0) (y)	Employment Rate (%)	Average Annual Income (1000 kwn)	Deaths (n)			Cost of Lost Productivity Due to Premature Death (1000 kwn)		Cost of Lost Productivity Due to Premature Death (1000 kwn)
				KCD I60-I69	Ischemic	Hemorrhagic	Ischemic	Hemorrhagic	Stroke
20~29 (25)	57.06	57.9	25,680	50	13	37	10,986	31,433	42,420
30~39 (35)	47.06	74.2	37,655	197	51	146	67,087	191,938	259,026
40~49 (45)	37.06	79.1	44,510	867	225	642	292,993	838,256	1,131,249
50~59 (55)	27.06	74.4	40,975	1,794	465	1,329	383,302	1,096,630	1,479,933
60~69 (65)	17.06	38.9	28,690	2,572	666	1,906	126,832	362,867	489,700
					1,419	4,061	881,203	2,521,126	3,402,329

Abbreviations: kwn, Korean won; KCD, Korean Standard Classification of Diseases.
